# Sex differences in gene expression with galactosylceramide treatment in *Cln3^Δex7/8^* mice

**DOI:** 10.1371/journal.pone.0239537

**Published:** 2020-10-02

**Authors:** Joelle Makoukji, Sally El-Sitt, Nadine J. Makhoul, Jihane Soueid, Humam Kadara, Rose-Mary Boustany

**Affiliations:** 1 Department of Biochemistry and Molecular Genetics, American University of Beirut Medical Center, Beirut, Lebanon; 2 Department of Translational Molecular Pathology, University of Texas MD Anderson Cancer Center, Houston, Texas, United States of America; 3 Neurogenetics Program, AUBMC Special Kids Clinic and Division of Pediatric Neurology, Department of Pediatrics and Adolescent Medicine, American University of Beirut Medical Center, Beirut, Lebanon; Universidad de Jaen, SPAIN

## Abstract

**Background:**

CLN3 disease is caused by mutations in the *CLN3* gene. The purpose of this study is to discern global expression patterns reflecting therapeutic targets in CLN3 disease.

**Methods:**

Differential gene expression in vehicle-exposed mouse brain was determined after intraperitoneal vehicle/Galactosylceramide (GalCer) injections for 40 weeks with GeneChip Mouse Genome 430 2.0 arrays.

**Results:**

Analysis identified 66 genes in male and 30 in female brains differentially expressed in GalCer-treated versus vehicle-exposed *Cln3*^*Δex7/8*^ mice. Gene ontology revealed aberrations of biological function including developmental, cellular, and behavioral processes. GalCer treatment altered pathways of long-term potentiation/depression, estrogen signaling, synaptic vesicle cycle, ErbB signaling, and prion diseases in males, but prolactin signaling, selenium compound metabolism and steroid biosynthesis in females. Gene-gene network analysis highlighted networks functionally pertinent to GalCer treatment encompassing motor dysfunction, neurodegeneration, memory disorder, inflammation and astrogliosis in males, and, cataracts, inflammation, astrogliosis, and anxiety in females.

**Conclusions:**

This study sheds light on global expression patterns following GalCer treatment of *Cln3*^*Δex7/8*^ mice. Understanding molecular effects of GalCer on mouse brain gene expression, paves the way for personalized strategies for treating this debilitating disease in humans.

## Introduction

Juvenile neuronal ceroid lipofuscinosis, now classified as CLN3 disease, represents the most common form of the neuronal ceroid lipofuscinoses (NCLs), a family of fatal inherited pediatric neurodegenerative disorders [1]. CLN3 disease is caused by mutations in the *CLN3* gene identified in 1995 by the International Batten Disease Consortium [1–3]. Clinical features of CLN3 disease include rapidly progressive blindness due to retinitis pigmentosa, epileptic seizures, psychomotor decline and premature death [1]. The presence of characteristic ultrastructural fingerprint inclusion bodies present in neurons and other cells has been described in cytoplasmic storage material [4]. Patient brain demonstrated elevated levels of the pro-apoptotic lipid second messenger, ceramide. The *CLN3* gene encodes a 48 kDa integral membrane protein implicated in endocytosis, endocytic trafficking [5–10], autophagy [11, 12], cell growth and survival [13, 14], apoptosis [14, 15], lysosomal pH regulation [16–18] and transport of galactosylceramide (GalCer) from Golgi to lipid rafts (LR) in plasma membrane (PM) [19]. The CLN3 protein harbors a highly conserved VYFAE motif necessary for its role in cell growth and apoptosis, discovered following detailed site-directed mutagenesis of the intact CLN3 protein. Molecular modeling of this motif placed it within a stretch of amino acids representing a structural GalCer lipid raft-binding domain [19].

*Cln3*-knockout (*Cln3*^*−/−*^) and *Cln3*^*Δex7/8*^ knock-in mice are commonly utilized mouse models of CLN3 disease [20]. In *Cln3*^*Δex7/8*^ knock-in mice, exons 7 and 8 were removed from the endogenous *Cln3* gene by targeted recombination creating a “knock-in” of exons 7/8-deleted. The latter mouse model mimics the most commonly prevalent human CLN3 mutation [21] and represents the most suitable model to test therapeutic strategies.

GalCer exerts a positive impact on cell growth and survival [22]. Knockdown of galactosylceramide transferase in normal cells results in diminished growth and increased apoptosis. Cell growth rate increases after supplementation with GalCer at a concentration of 50 ng/mL. A short-term study of injecting GalCer in homozygous *Cln3*^*Δex7/8*^ mice for a period of 17 weeks resulted in significant improvements in the course of CLN3 disease including reduced ceramide levels, subunit C accumulation and gliosis in specific brain regions. This led to a prolonged trial of 40 weeks with exogenous GalCer that demonstrated beneficial effects on neurobehavioral and biochemical parameters, and increase in longevity of homozygous *Cln3*^*Δex7/8*^ mice [23].

Recently, microarray gene expression analysis of Cb*Cln3*^*Δex7/8*^ and Cb*Cln6*^*nclf*^ cerebellar cells showed that *Cln3* and *Cln6* mutations initiate disease via distinct molecular and cell biological processes that converge on a common pathway [5]. So far, our knowledge of brain expression profiling of *Cln3*^*Δex7/8*^ mice is lacking. In the present study, candidate genes which may be involved in the neuroprotective mechanisms of GalCer treatment were identified by comparing gene expression in the brains of *Cln3*^*Δex7/8*^ mice. The purpose of the present work was to elucidate differences between the brains of *Cln3*^*Δex7/8*^ mice treated with GalCer and *Cln3*^*Δex7/8*^ control mice treated with vehicle. To determine this, we performed a microarray analysis. Gene expression data can accurately define the consistent gene expression schematic of the brain transcriptome and can provide insights into the molecular mechanism of action of GalCer. The analysis revealed statistically significant differentially expressed genes and pathways, including anti-inflammatory and neuroprotective mechanisms, and topologically functional gene-gene networks that may explain the positive therapeutic effects observed in GalCer-treated *Cln3*^*Δex7/8*^ mice. This study lays the foundation to identify potential mechanisms that may aid in development of personalized therapeutic strategies for human CLN3 disease.

## Materials and methods

### Animals and housing conditions

Experiments were carried out in homozygous *Cln3*^*Δex7/8*^ mice bred on a C57BL/6J background, (Jackson laboratories-Bar Harbor, ME, US). Animals were housed in standard cages in a controlled environment with a temperature of 22–24°C, humidity of 60% and a 12-hour light-dark cycle. Food and water were provided ad libitum. The experiments took place in the animal care facility of the American University of Beirut [AUB assurance identification # F16-00328, Office of Laboratory Animal Welfare (OLAW)]. All animal experiments were performed in accordance with AUB Institutional Animal Care and Use Committee (IACUC) guidelines according to an approved IACUC protocol # 17-03-RN407. Four homozygous *Cln3*^*Δex7/8*^ males and five *Cln3*^*Δex7/8*^ females were bred in the AUBMC animal care facility to obtain the total number of mice needed (n = 16).

### Experimental design

For sequential steps, experimental procedures and number of animals in each experimental group see S1 Fig. Male and female *Cln3*^*Δex7/8*^ mice were treated with vehicle (n = 8) or GalCer (n = 8). The injection was given intraperitoneally, six days/week, for a period of 40 weeks. The genotypes were confirmed by polymerase chain reaction (PCR) on DNA extracted from mouse tail blood. Mice were checked for weight, coat appearance and overall cage activity to monitor their general health.

### Galactosylceramide treatment

Pharmacological treatment of all animals was started at the age of four weeks. Mice were subjected to GalCer/vehicle long-term treatment for a period of 40 weeks. The concentration of GalCer used was 1 mg/kg; D-Galactosyl-β1–1’-N-Dodecanoyl-D-erythro-Sphingosine [C12 α 1-D-galactosyl ceramide; Avanti], dissolved in 0.6% DMSO/0.15% polysorbate 20 in PBS.

### Brain tissue sampling for RNA extraction

Animals were anesthetized with ketamine/xylazine cocktail and brains were rapidly dissected and “snap” frozen in liquid nitrogen to preserve RNA integrity, and stored at -80°C. Total RNA was isolated from 60–65 mg of fresh brain tissue using the mirVana™ miRNA Isolation Kit (Thermo Fisher Scientific, MA, USA) that allows isolation of total RNA with excellent yields, following manufacturer protocols. RNA extraction was followed by RNA cleanup using RNeasy Plus Mini Kit (Qiagen, Germany) following manufacturer instructions, and stored at -80°C. RNA integrity was assessed using the Experion™ Automated Electrophoresis System (BioRad, CA, USA). RNA concentrations were determined by absorption at 260 nm wavelength as well as RNA purity by checking A_260_/A_280_ and A_260_/A_230_ ratios, with a ND-1000 spectrometer (Nanodrop Technologies LLC, DE, USA).

### Microarray gene expression profiling in mice

**Fig 1:**
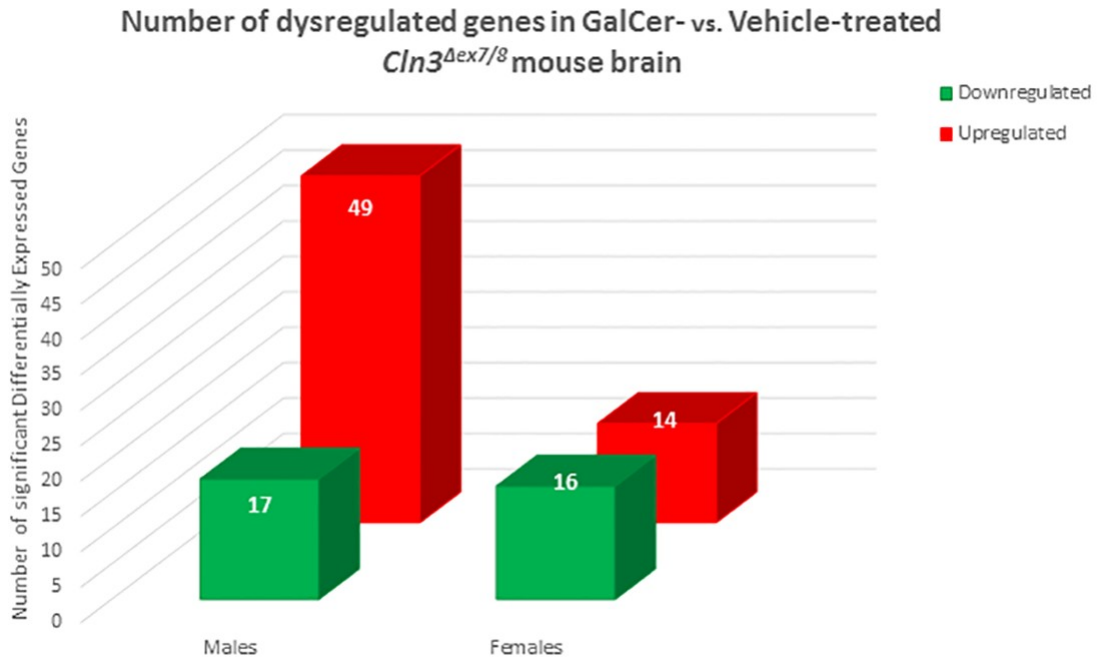
GalCer induces sex-specific alterations in gene expression in *Cln3*^*Δex7/8*^ mouse brain. Bar chart indicating differences in numbers of significant differentially expressed genes affected by GalCer compared to vehicle treatment in *Cln3*^*Δex7/8*^ male and female mice. A cut-off p-value < 0.05 and fold-change ≥ ±1.3 were assumed to identify genes significantly modulated by GalCer treatment.

Microarray analysis of 16 *Cln3*^*Δex7/8*^ brain tissue samples treated with vehicle or GalCer (four males and four females in each group) was performed using the GeneChip Mouse Genome 430 2.0 array platform from Affymetrix (Affymetrix Inc., CA, USA) representing over 45,000 probe sets) (Fig 1). Samples were prepared and microarrays processed using the GeneChip™ 3’ IVT PLUS Reagent Kit (Thermo Fisher Scientific, MA, USA) according to manufacturer instructions. Briefly, 100 ng of total RNA were labeled, fragmented, and then hybridized to arrays. After washing and staining using GeneChip Fluidics Station 450 (Thermo Fisher Scientific, MA, USA), arrays were scanned with the GeneChip Scanner 3000 7G (Thermo Fisher Scientific, MA, USA). Cell Intensity Data (CEL) files were generated with the Affymetrix GeneChip™ Command Console (AGCC) software version 3.2 (Thermo Fisher Scientific, MA, USA).

### Data analysis

Differentially expressed genes in groups were identified (Partek Genomics Suite software version 7.0- Partek, MO, USA). Probe set data were summarized and background adjusted using the Robust Multi-Array Average (RMA) algorithm [24]. All data was normalized using non-linear transformation termed Quantile Normalization, and further filtered to remove noise and extreme expression values. The false discovery rate (FDR) was not applied to the microarray data after statistical testing, because there were no genes that met a FDR p-value < 0.05 for the GalCer treatment effect in both genders. To overcome this issue, dysregulated genes identified by microarray analysis were ultimately evaluated by a gold standard experimental method (quantitative real time transcription—polymerase chain reaction (qRT-PCR)). In this circumstance, it is acceptable not to apply FDR correction, and examine a larger pool of differentially expressed genes.

Two-way analysis of variance (ANOVA) led to detection of significant differences of treated groups taking into account gender as a variable or factor. Contrasts (treatment and gender) were included in the model based on the comparison of interest. All further sub-lists were created using genes that passed the ANOVA p-value and fold change threshold. A gene by gene p-value < 0.05 was considered significant, and an arbitrary threshold of 1.3-fold difference was applied to discern differentially modulated genes. Partek Genomics Suite and Partek Pathway^™^ version 7.0 (Partek, MO, USA) were used for cluster analysis and for analysis of gene ontology (GO) and pathways. Networks of biologically related genes were created using Pathway Studio version 11.4 (Ariadne Genomics, MD, USA).

### Quantitative real-time PCR

Microarray results were confirmed by qRT-PCR. Total RNA extracted from fresh brain tissue was reverse transcribed using RevertAid Reverse Transcriptase (Thermo Fisher Scientific, MA, USA) with 2 μg of input RNA and random primers (Thermo Fisher Scientific, MA, USA). qRT-PCR reactions were performed in 384-well plates using specific primers (Tm = 60°C) (TIB MOLBIOL, Germany) (see S1 Table) and the iTaq SYBR Green Supermix (BioRad, CA, USA) as a fluorescent detection dye, in CFX384TM Real-Time PCR (BioRad, CA, USA), in a final volume of 10 μl. To characterize generated amplicons and to control contamination by unspecific by-products, melt curve analysis was applied. Each reaction was performed in triplicate. All results were normalized to *Gapdh* mRNA level and calculated using the ΔΔCt method.

### Statistical analysis

Brain mRNA expression was quantified in GalCer-treated compared to vehicle-treated *Cln3*^*Δex7/8*^ mice. Continuous data was expressed as means ± SEM, and compared by the two-tailed Student’s *t-*test. Gene expression data derived by Affymetrix or qRT-PCR was compared in brain from GalCer- and vehicle-treated *Cln3*^*Δex7/8*^ mice by standard Student’s *t*-test.

GraphPad Prism 6 (GraphPad Software, CA, USA) was used for statistical analysis. Affymetrix expression data was analyzed using Partek Genomics Suite and Partek Pathway^™^ version 7.0 (Partek, MO, USA). All tests were two-sided and a p-value < 0.05 considered statistically significant.

## Results

### Transcriptional profiles of male/female *Cln3*^*Δex7/8*^ brain treated with GalCer

**Fig 2:**
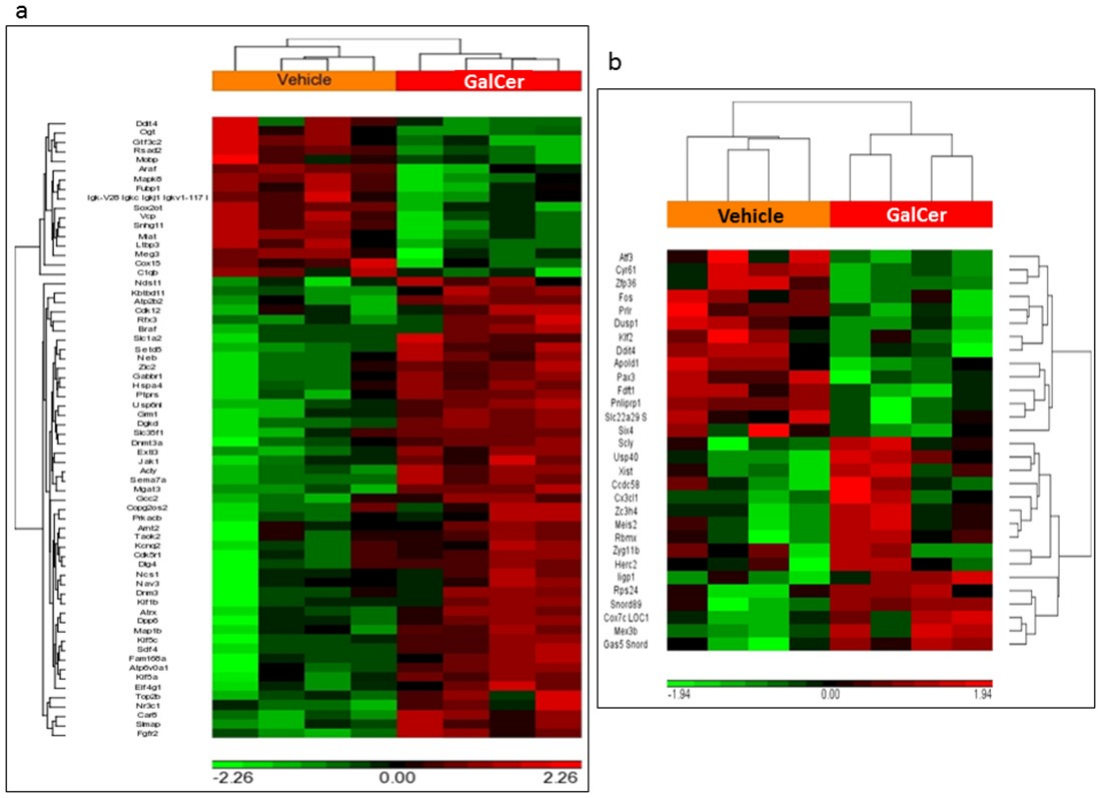
Differentially expressed genes in GalCer versus vehicle-treated male and female *Cln3*^*Δex7/8*^ mouse brain. 2-dimentional heat maps of (a) 66 DEGs in male *Cln3*^*Δex7/8*^ mice, and (b) 30 DEGs in female *Cln3*^*Δex7/8*^ mice. Heat maps show mRNA abundance intensities of differentially expressed genes in profiled samples. Robust Multi-Array Average (RMA) preprocessed data is transformed to z-scores. The legend represents relative over- (red) and under-expression (green). Labeling at the top represents Vehicle control samples and GalCer-treated samples. A cut-off p-value < 0.05 and fold-change ≥ ±1.3 were assumed to identify genes significantly modulated by GalCer treatment.

**Fig 3:**
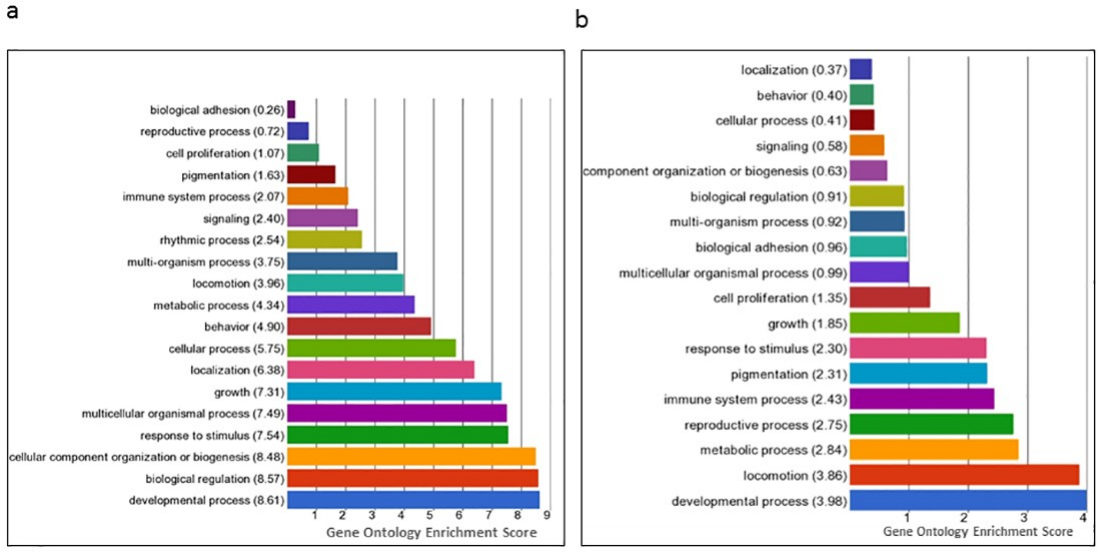
Bar chart of GO enrichment scores for DEGs according to biological processes in male/female *Cln3*^*Δex7/8*^ mouse brain. Gene expression in GalCer- versus vehicle-treated male (a) and female (b) *Cln3*^*Δex7/8*^ mouse brain. Differential expression is assumed with a p-value < 0.05, and a gene count > 2 of the DEGs. Functional categories of DEGs were obtained using GO annotations from the Partek Genomics Suite classification system.

Transcriptomes of brain tissue of *Cln3*^*Δex7/8*^ mice treated with GalCer or vehicle were compared using the Affymetrix Mouse Genome platform. Identification of differentially expressed genes (DEGs) in brain from GalCer- and vehicle-treated *Cln3*^*Δex7/8*^ mice occurred. Assumption of a cut-off p-value < 0.05 and fold-change ≥ ±1.3 identified genes significantly modulated by GalCer. Using a two-way ANOVA model, male mice had 66 DEGs (49 upregulated and 17 downregulated genes) modulated in brain tissue from GalCer-treated mice compared with samples from vehicle exposed male animals. Analysis of female mice revealed 30 DEGs (14 upregulated and 16 downregulated genes) modulated by GalCer treatment (Fig 1). The DEGs for each group (males and females) are depicted in S2–S4 Tables. Fig 3 shows two-way hierarchical clustering (2D-heatmap) of the DEGs in male (Fig 2a) and female (Fig 2b) *Cln3*^*Δex7/8*^ mouse brains.

### Functional gene ontology analysis of differentially expressed genes in male/female *Cln3*^*Δex7/8*^ brain

**Fig 4:**
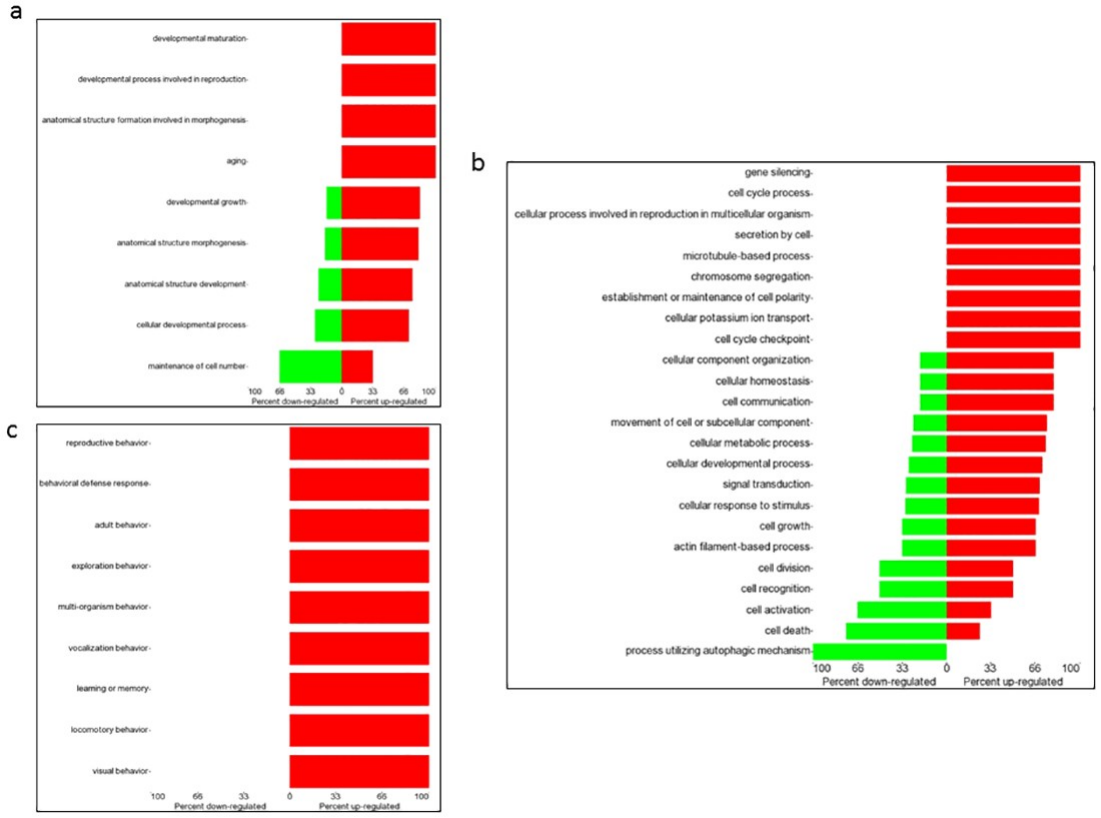
Forest plot of GO for DEGs in biological processes in male *Cln3*^*Δex7/8*^ mouse brain. Gene expression in GalCer- versus vehicle-treated *Cln3*^*Δex7/8*^ male mouse brain. The criterion for differential expression assumes a p-value < 0.05. Forest plots of biological processes including (a) developmental, (b) cellular, and (c) behavioral processes depict percentage of down- (green) or up-regulated (red) genes in each category.

**Fig 5:**
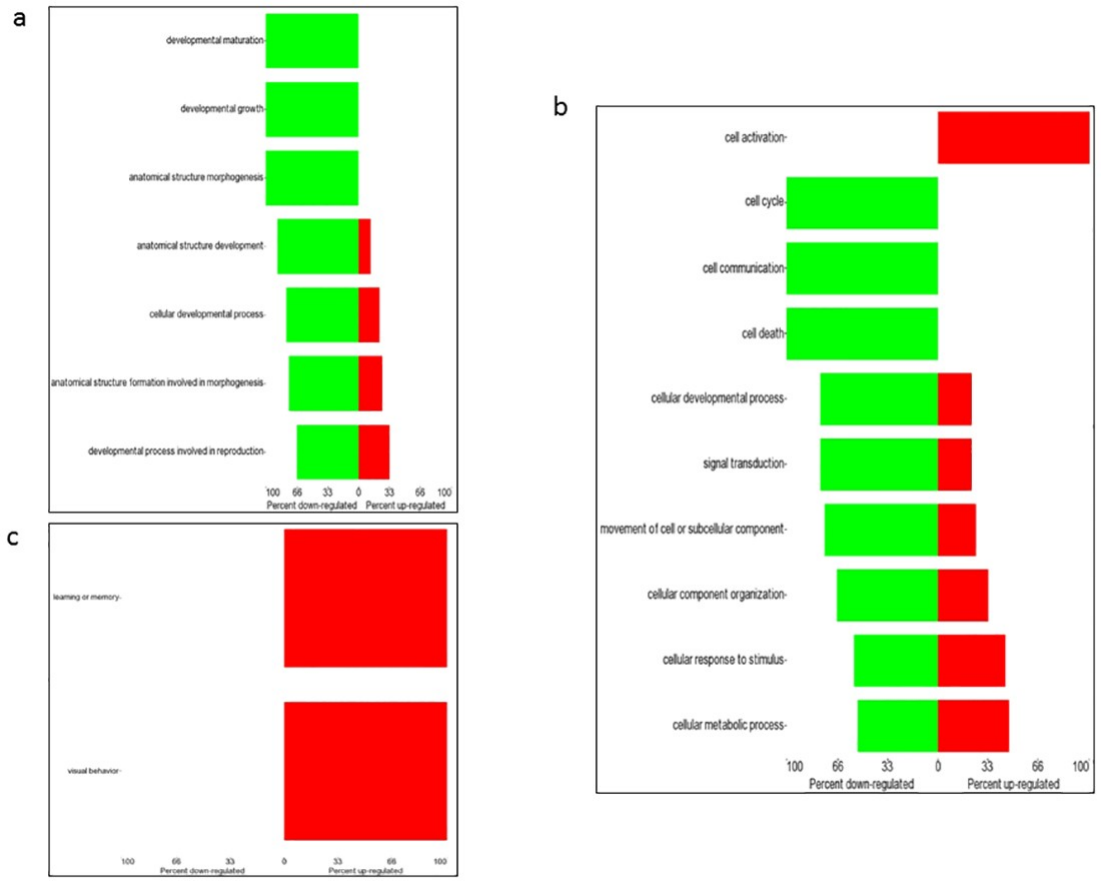
Forest plot of GO for DEGs in biological processes in female *Cln3*^*Δex7/8*^ mouse brain. Gene expression in GalCer versus vehicle-treated female *Cln3*^*Δex7/8*^ mouse brain. The criterion for differential expression assumes a p-value < 0.05. Forest plots of biological processes of (a) developmental, (b) cellular, and (c) behavioral processes depict percentage of down- (green) or up-regulated (red) genes in each category.

Functional and biological annotation of identified DEGs by gene ontology (GO) analysis using the Partek Genomics Suite platform was carried out. A cut-off p-value < 0.05 and a gene count > 2 was used to enrich GO terms. The results of the GO analysis indicated that altered transcription of genes in male and female GalCer-treated *Cln3*^*Δex7/8*^ mouse brain included genes involved in developmental, cellular and behavioral processes (Fig 3a and 3b). In GalCer-treated versus vehicle-treated males, upregulated genes in the brain of *Cln3*^*Δex7/8*^ mice were involved in developmental growth (*Atrx*, *Fgfr2*, *Map1b*, *Meg3*, *Sema7a*, *Slc1a2*), cell cycle (*Top2b*, *Atrx*), cell growth (*Sema7a*, *Map1b*, *Fubp1*), exploration behavior (*Dlg4*), learning or memory (*Braf*), locomotor behavior (*Atp2b2*, *Dlg4*, *Grm1*), and visual behavior (*Braf*, *Slc1a2*). Downregulated genes in male *Cln3*^*Δex7/8*^ mouse brain were associated with cell death (*Ogt*, *Mapk8*, *Ddit4*), and autophagy (*Vcp*) (Fig 4a–4c). In females, GalCer upregulated genes in *Cln3*^*Δex7/8*^ mouse brain were associated with cell activation (*Cxcl1*), learning or memory, and visual behavior (*Meis2*) compared to vehicle-treated mice. Downregulated genes in female *Cln3*^*Δex7/8*^ mouse brain tissue included genes involved in modulation of developmental growth (*Klf2*, *Prlr*), and cell death (*Cyr61*) (Fig 5a–5c).

### Major pathways modulated by GalCer in male/female *Cln3*^*Δex7/8*^ brain

Analysis of major molecular pathways in response to GalCer treatment involved determining differentially regulated genes in male and female *Cln3*^*Δex7/8*^ brain using the Kyoto Encyclopedia of Genes and Genomes (KEGG) Pathway database in Partek. The most statistically significant candidate pathways of interest in male *Cln3*^*Δex7/8*^ mice were “long-term potentiation”, “long-term depression”, “estrogen signaling pathway”, “synaptic vesicle cycle”, “ErbB signaling pathway”, and “prion diseases”. All significant canonical pathways identified in male *Cln3*^*Δex7/8*^ mouse brain appear in S5 Table. Upregulated genes involved in these pathways included *Grm1*, *Prkacb*, *Braf*, *Jak1*, *Gabbr1*, *Dnm3* and *Atp6v0a1* (S2 Table). Downregulated genes comprised *Mapk8* and *C1qb* (S3 Table). In female *Cln3*^*Δex7/8*^ mouse brain, significant pathways included “prolactin signaling pathway”, “selenium compound metabolism”, and “steroid biosynthesis” (S4 Table). An upregulated gene involved in these pathways is *Scly*. Downregulated genes are *Fos*, *Prlr* and *Fdft1* (S4A and S4B Table). These findings suggest that GalCer treatment leads to up- and down-regulation of gene expression in brain tissue of *Cln3*^*Δex7/8*^ mice, affecting metabolism and signaling pathways.

**Fig 6:**
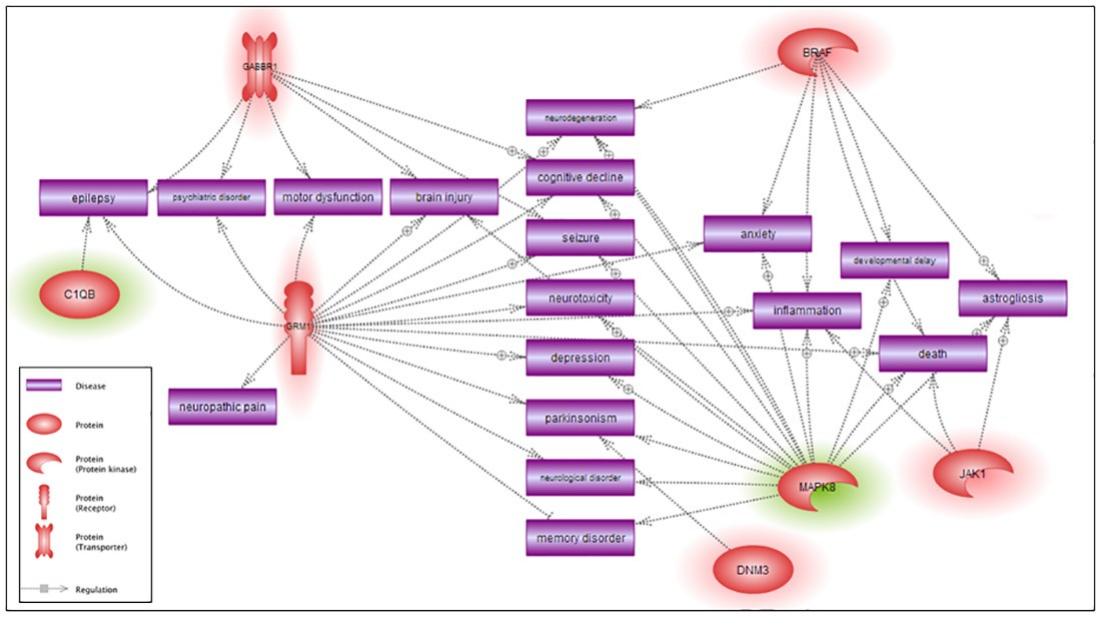
Gene-disease relationship network of significant DEGs in GalCer- versus vehicle-treated male *Cln3*^*Δex7/8*^ mice. Pathway Studio software generated network interactions between diseases and most significant DEGs in male *Cln3*^*Δex7/8*^ mouse brain (p-value < 0.05, stringency ≥ ± 1.3 fold-change in expression). Depiction of genes is by their gene symbols. Upregulated genes are highlighted with a red halo and downregulated genes are highlighted with a green halo. Indirect regulation is indicated by dotted gray lines.

**Fig 7:**
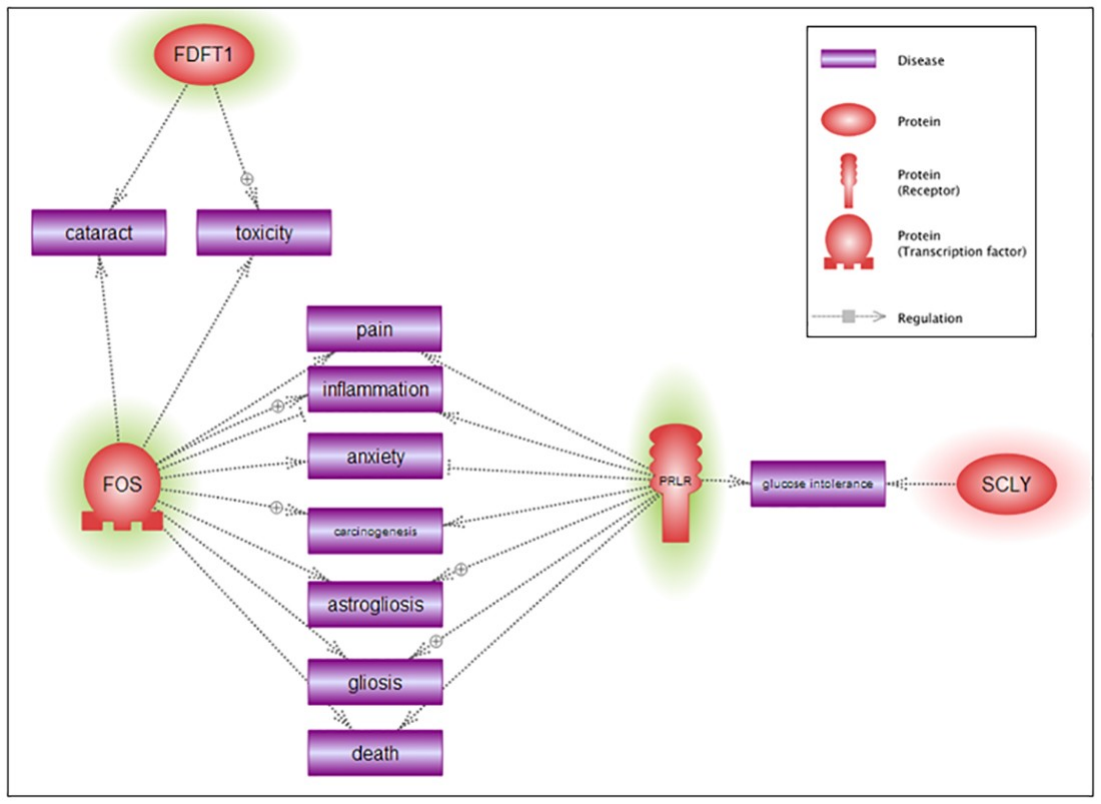
Gene-disease relationship network of significant DEGs in GalCer- versus vehicle-treated female *Cln3*^*Δex7/8*^ mice. Pathway Studio software generated network interactions between diseases and most significant DEGs in female *Cln3*^*Δex7/8*^ mouse brain (p-value < 0.05, stringency ≥ ± 1.3 fold-change in expression). Depiction of genes is by gene symbols. Upregulated genes are highlighted with a red halo and downregulated genes are highlighted with a green halo. Indirect regulation is indicated by dotted gray lines.

DEGs were then topologically organized into functional gene-gene interaction networks in male (Fig 6) and female (Fig 7) *Cln3*^*Δex7/8*^ mice. The putative disease and disorders identified in brain of *Cln3*^*Δex7/8*^ mice included motor dysfunction, epilepsy, neurodegeneration, cognitive decline, seizures, depression, memory disorder, inflammation, astrogliosis, and developmental delay in males, and, cataracts, inflammation, astrogliosis, anxiety, and glucose intolerance in females.

### Validation of mRNA microarray analysis results by qRT-PCR

**Fig 8:**
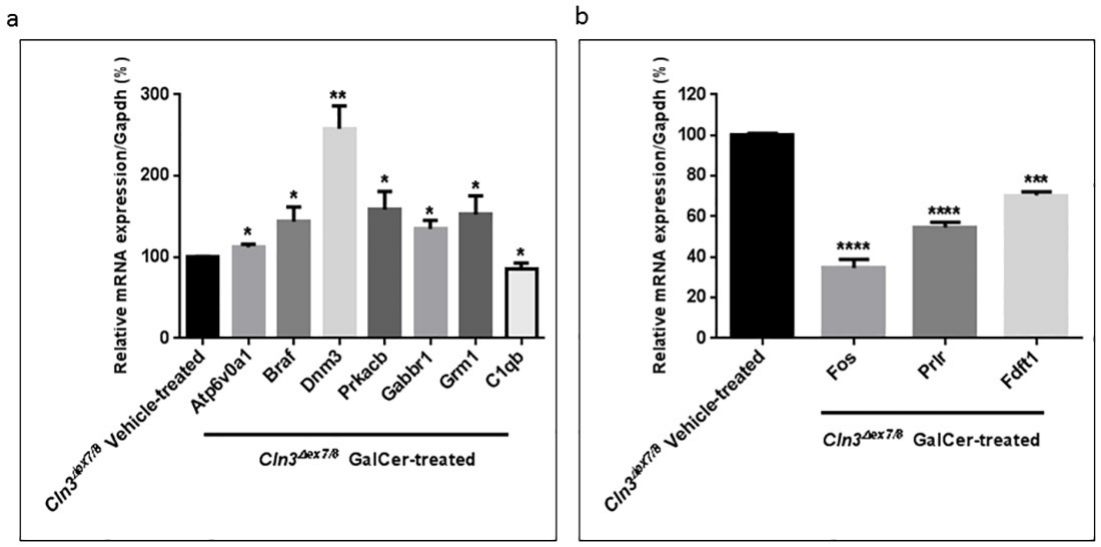
Validation of microarray analysis results with qRT-PCR in male and female *Cln3*^*Δex7/8*^ mouse brain. Relative mRNA expression measured by qRT-PCR. (a) The following differentially expressed genes in male *Cln3*^*Δex7/8*^ mouse brains were randomly chosen: *Atp6v0a1*, *Braf*, *Dnm3*, *Prkacb*, *Gabbr*, *Grm1*, and *C1qb*. Values are means of the fold changes normalized to *Gapdh* mRNA expression, with standard errors represented by vertical bars.*p-value < 0.05 and ** p-value < 0.01 by Student’s *t*-test (n = 4). (b) The following differentially expressed genes in female *Cln3*^*Δex7/8*^ mouse brains were randomly chosen: *Fos*, *Prlr*, and *Fdft1*. Values are means of the fold changes normalized to *Gapdh* mRNA expression, with standard errors represented by vertical bars. *** p-value < 0.001 and **** p-value < 0.0001 by Student’s *t*-test (n = 4).

Ten genes were randomly chosen for further analysis by qRT-PCR based on biological relevance. Results exhibit consistency with microarray analysis, validating findings. GalCer treatment induced significant increases in expression levels of *Atp6v0a1* (p-value < 0.05), *Braf* (p-value < 0.05), *Dnm3* (p-value < 0.01), *Prkacb* (p-value < 0.05), *Gabbr1* (p-value < 0.05), and *Grm1* (p-value < 0.05) in male *Cln3*^*Δex7/8*^ mouse brain compared with corresponding levels in vehicle-treated male *Cln3*^*Δex7/8*^ mouse brain. Attenuated expression of *C1qb* (p-value < 0.05) in male *Cln3*^*Δex7/8*^ mice, and *Fos* (p-value < 0.0001), *Prlr* (p-value < 0.0001), and *Fdftt1* (p-value < 0.001) in female *Cln3*^*Δex7/8*^ GalCer-treated mouse brain compared to corresponding samples from vehicle-treated *Cln3*^*Δex7/8*^ mice (Fig 8a and 8b). The data sets obtained from microarray analysis accurately reflect differential gene expression in brain of *Cln3*^*Δex7/8*^ mice treated with GalCer or vehicle.

## Discussion

Several therapeutic approaches have been developed for treatment of CLN3 disease including anti-inflammatory agents, immunosuppressants and gene replacement therapies [3]. One of the pathological features of CLN3 disease is the increase in ceramide levels noted in brains of CLN3 and CLN2 disease patients, previously referred to as juvenile and late-infantile NCL, respectively [25]. The therapeutic approach in this study consisted of treating *Cln3*^*Δex7/8*^ mice with daily injections of GalCer over 40 weeks. GalCer supplementation resulted in promising outcomes improving some neurobehavioral aspects of the disease, increasing longevity of male and female mice, in addition to reducing ceramide levels in homozygous *Cln3*^*Δex7/8*^ mouse brain from males and females [23].

Transcriptomic profiling analysis allowed gaining insight into molecular mechanisms potentially underlying the effects of GalCer treatment in brains of *Cln3*^*Δex7/8*^ mice. Distinct microarray expression profiles were identified for male and female *Cln3*^*Δex7/8*^ mice, and results were validated by qRT-PCR analysis of selected genes. Overall, findings suggest that GalCer treatment can markedly influence *Cln3*^*Δex7/8*^ brain gene expression, in a sex-specific manner.

KEGG pathway analysis identified long-term potentiation (LTP) and long-term depression (LTD) to be impacted by GalCer treatment with high statistical significance, and 3 differentially expressed genes associated with these pathways. Significant increases in mRNA expressions of *Grm1*, *Prkacb* and *Braf* related to the LTP and LTD occurred in GalCer-treated male *Cln3*^*Δex7/8*^ mouse brain (S2 Table). Long-term synaptic plasticity is related to be the molecular basis of learning and memory [26]. LTP and LTD are two forms of synaptic plasticity demonstrated to occur in most brain regions, particularly the cerebellum [27]. L-glutamate is the major excitatory neurotransmitter in the central nervous system and activates ionotropic and metabotropic glutamate receptors like glutamate receptor, metabotropic 1 (*Grm1*). Glutamatergic neurotransmission is involved in many aspects of normal brain function and can be perturbed in schizophrenia, bipolar disorder, and depression. Zhang *et al*. demonstrated expression of several genes encoding glutamatergic and non-glutamatergic receptors related to LTP and LTD to be reduced in cerebellum of mice exposed to arsenic: *Grm1* gene expression, among others, induces neurotoxicity and impairment of learning and memory [28]. The second gene, cAMP-dependant protein kinase catalytic subunit beta (*Prkacb*), encodes a catalytic subunit isoform of the protein kinase A holoenzyme (PKA). *Prkacb* phosphorylates the transcription factor, cAMP-response element-binding protein (CREB), which regulates the expression of several other genes [29]. Lower expression of *Prkacb* in the prefrontal cortex occurs in major depression [30]. The third gene, *Braf*, is a pro-survival gene and a regulator of cellular growth and homeostatic turnover. It codes for the protein B-RAF, a serine/threonine kinase, which is part of the MEK/ERK pathway. This pathway regulates a variety of important intracellular functions including cellular proliferation, and physiologic cell death [31]. Schroeder *et al*. suggested that control of *Braf* expression may be an important determinant in the apoptosis-related physiology of bipolar illness, leading to brain tissue loss in bipolar patients [32]. Yuan *et al*. found a deficit in B-RAF protein expression in postmortem cortex of bipolar subjects [33]. Correcting the *Braf* abnormality with neuroprotective mood stabilizers that activate the MEK/ERK pathway, could allow for normalization of cell survival/death pathways [34]. In the present study, exogenous GalCer significantly increased expression levels of *Grm1*, *Prkacb*, and *Braf* in *Cln3*^*Δex7/8*^ male mouse brains (S2 Table), which if low, may contribute to the underlying neuropathobiology in male *Cln3*^*Δex7/ 8*^ mice.

GalCer significantly regulated the estrogen signaling pathway, while increasing expression of the Gamma-Aminobutyric Acid (GABA) Type B Receptor Subunit 1 (*Gabbr1)* gene (S2 Table). GABA acts as the main inhibitory neurotransmitter in the central nervous system (CNS). GABA receptor types include GABA_A_, GABA_B_, and GABA_C_. GABA_B_ receptors are heterodimeric and consist of two subunits, GABBR1 and GABBR2. GABA_B_ abnormalities are implicated in autism [35], bipolar disorder [36], major depression [37], and schizophrenia [36]. Reductions in GABBR1 and GABBR2 proteins are observed in prefrontal cortex of subjects with bipolar disorder [36]. Also, significant reductions in *Gabbr1* and *Gabbr2* gene expression were reported to be present in lateral cerebellum of subjects with schizophrenia, bipolar disorder, and major depression, providing further evidence of GABAergic dysfunction in these diseases [38]. Chronic administration of antidepressants increases expression of GABA_B_ receptors in brain of major depression animal models [39]. In this study, exogenous GalCer significantly upregulated *Gabbr1* expression in *Cln3*^*Δex7/8*^ male mouse brain, a desirable outcome decreasing severity of disease in affected male mice.

Expression of genes involved in synaptic vesicle cycle pathways is affected by exogenous GalCer, with increased expression of *Dnm3* and *Atp6v0a1* genes in male *Cln3*^*Δex7/8*^ mouse brain (S2 Table). Dynamin is a super-family of large GTPase proteins that polymerize during biological activity. Dynamin polymerization in membrane fission plays a significant role in synapses suggestive of a relationship between synapses and dynamins [40]. Several animal model studies show that *Dnm1* and *Dnm3* genes are highly expressed in neurons [41]. The role of *Dnm3* in neurodegenerative diseases is unknown. Intracellular vesicles acidify by recruitment and activation of the vacuolar ATPase (v-ATPase) complex, which pumps protons into the lumen of vesicles, lowering their pH [42]. ATPase H+ Transporting V0 Subunit A1 (Atp6v0a1), a subunit of v-ATPase, mediates acidification of eukaryotic intracellular organelles, necessary for intracellular processes of receptor-mediated endocytosis, and synaptic vesicle proton gradient generation. v-ATPase subunits have other functions in cells besides vesicle acidification. In microglial cells in the CNS of zebrafish, disruption of *Atp6v0a1* causes failure of auto-phagosomes to mature and fuse, but they acidify normally [43]. The relevance of *Atp6v0a1* disruption to neurodegenerative diseases is not clear. In this study, exogenous GalCer significantly increased expression levels of *Dnm3* and *Atp6v0a1* in male *Cln3*^*Δex7/8*^ mouse brain, suggesting a new avenue for study and treatment of neurodegenerative diseases (S2 Table).

Also, the ErbB signaling pathway and the *Mapk8* gene itself were significantly modulated by exogenous GalCer (S3 Table). The c-Jun N-terminal kinases (JNKs), a subfamily of MAP kinases (MAPK), are central signal transducers in the mammalian brain, mostly associated with pathogenesis and neuronal death in neurodegenerative diseases [44]. De Lemos *et al*. show that absence of JNK isoforms confers neuroprotection against neuronal damage induced by kainic acid, a potent neurotoxic agent able to induce status epilepticus, neuronal damage and gliosis in the hippocampus. Other studies demonstrated that lack of JNK isoforms is related to neuroprotection in epilepsy, Parkinson’s disease, ischemia, and Alzheimer’s disease [45–47]. In this study, GalCer significantly decreased the expression of *Mapk8* in male *Cln3*^*Δex7/8*^ mouse brain, possibly conferring neuroprotection (S3 Table).

Prion diseases or transmissible spongiform encephalopathies are fatal neurodegenerative conditions in humans and animals that originate spontaneously, genetically or by infection [48]. Terminal complement activation is one of the pathways leading to neuronal death as demonstrated in human prion disease [49]. Neuronal apoptosis is accompanied by elevation in C1q protein (*C1qa* and *C1qb* mRNA levels), reflecting degeneration of synapses [50]. In *Cln3*^*Δex7/8*^ male mice, expression of *C1qb* is downregulated in response to GalCer, perhaps as an attempt to reduce neuronal apoptosis (S3 Table). Of note, the structural GalCer binding motif VYFAE [13], specific to apoptosis in human CLN3 protein and a binding site for lipid rafts, defines a domain common to the V3 loop of the HIV-1 gp120 envelope protein, β-amyloid protein, and the infectious form of prionic protein [19].

Expression of *Prlr* and *Fos* was downregulated following GalCer administration in *Cln3*^*Δex7/8*^ females, indicating potential reduction of neuronal apoptosis (S4 Table). Anterior pituitary cell turnover occurring during the female sexual cycle involves complex regulation of cell proliferation and apoptosis by prolactin (PRL). PRL mediates its multiple functions through the prolactin receptor (PRLR) which is expressed as one long (PRLR_long_) and three short (PRLR_short_) isoforms in mice [51]. In rats, the PRL surge that occurs at pro-estrus coincides with a high apoptotic rate, suggesting that PRL exerts a direct anti-proliferative and pro-apoptotic effect on anterior pituitary cells [52]. The canonical signaling cascade of the PRLR, namely the Jak2/STAT5 cascade, leads to pro-apoptotic responses by regulating expression of Bcl-2 family proteins [53]. Proto-oncogene, c-Fos, is distributed in hippocampi, amygdala and piriform cortex of rat brain. Seizure activity, cortical brain injury and depression induce rapid and transient expression of c-Fos in hippocampal structures. Zhang *et al*. suggested that miR-129 could inhibit occurrence and development of epilepsy by repressing c-Fos expression via inhibition of the MAPK signaling pathway [54]. Selenocysteine lyase (*Scly*) mediates selenocysteine decomposition in the seleno-compound metabolism pathway [55]. Selenium is a necessary trace element with antioxidant properties essential for normal brain development and male fertility. Male mice lacking two key genes involved in selenium metabolism (*Scly*^-/-^ *Sepp1*^-/-^ mice), selenoprotein P (*Sepp1*) and selenocysteine lyase (*Scly*), develop severe neurological dysfunction, neurodegeneration, and audiogenic seizures manifesting in early adulthood [56]. Castration of these male mice increases brain seleno-protein levels, preventing behavioral deficits, attenuating neurodegeneration, and rescuing maturation of GABAergic inhibition. In this study, GalCer treatment of female *Cln3*^*Δex7/8*^ mice upregulates brain expression of the *Scly* gene, probably contributing to the attenuation of neurodegeneration in this disease (S4 Table).

Cholesterol is required for steroid hormone and bile acids biosynthesis, cell membrane organization, LR formation and maintenance. All are involved in multiple brain functions including growth factor signaling, synaptic transmission, and axon guidance. Impaired cholesterol metabolism in the brain is linked to many neurodegenerative diseases: Parkinson’s disease, Huntington’s disease, Alzheimer’s disease, Niemann-Pick type C disease and Smith-Lemli Opitz syndrome [57]. Alterations of lipid composition and stoichiometry of lipid rafts by depletion of cholesterol could serve as a therapeutic strategy for these disorders [58, 59]. In CLN3 disease, the sphingolipid composition of lipid rafts to that in Golgi and endoplasmic reticulum (LR/Golgi+ER) is altered [22]. Characterizing the impact of cholesterol level modulation in CLN3-deficient cells may be a therapeutic strategy worth exploring. The incidence of Alzheimer’s disease may be reduced by use of cholesterol-reducing agents like HMG-CoA reductase inhibitors [59]. In the female *Cln3*^*Δex7/8*^ mouse model, GalCer reduced expression of the squalene synthase gene *Fdft1*, which catalyzes the first step in sterol biosynthesis [60], suggesting studying the impact of this reduction on some of the neuropathological aspects of CLN3 disease is worthwhile (S4 Table).

**Fig 9:**
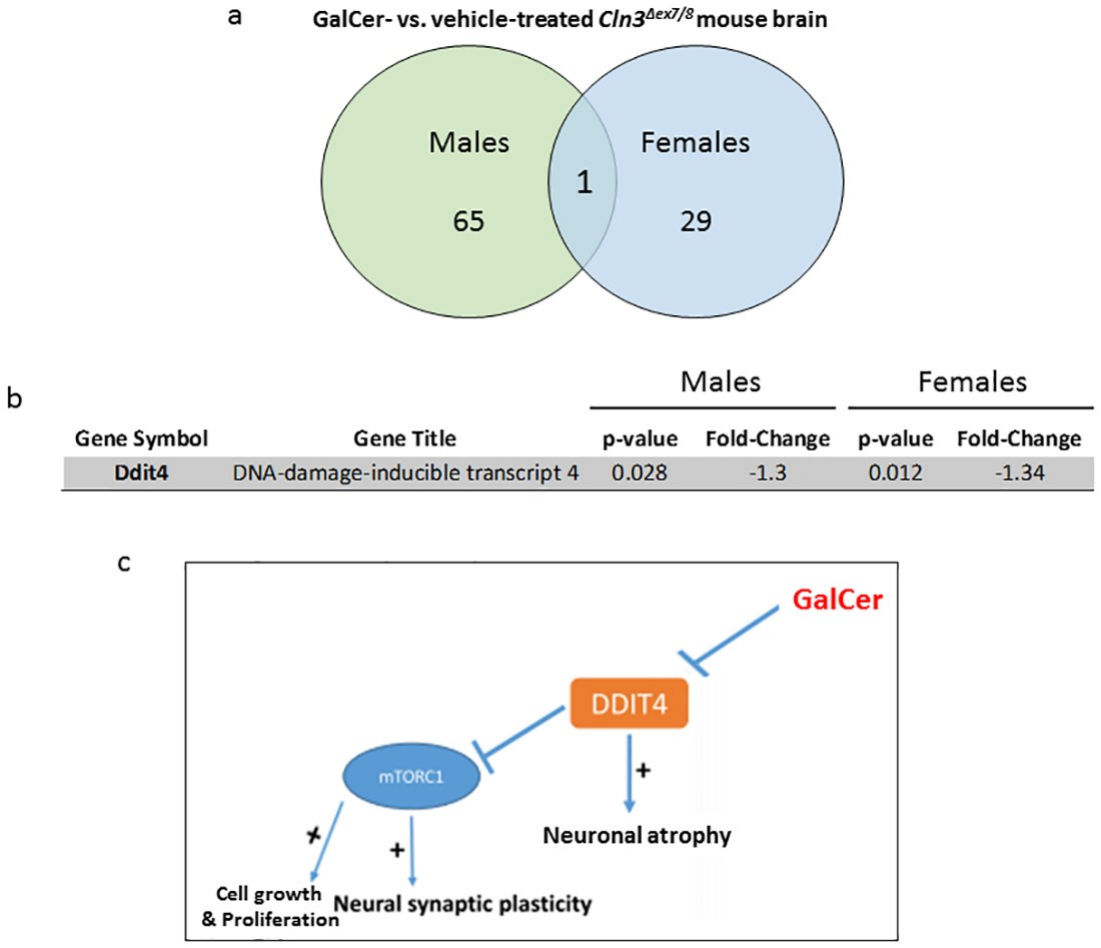
Comparative analysis of DEGs in male and female *Cln3*^*Δex7/8*^ mouse brain. Depiction of DEGs selected in male and female *Cln3*^*Δex7/8*^ mouse brain based on gene expression in GalCer compared to vehicle-treated *Cln3*^*Δex7/8*^ mice of both genders. The criterion for differential expression was a p-value < 0.05, and a stringency ≥ ± 1.3-fold change in expression. (a) A total number of 96 genes, with 65 and 29 genes exclusive for male and female *Cln3*^*Δex7/8*^ mouse brain, respectively, shown. One gene appears in the overlapping region, common to both datasets. (b) Fold change and p-value of the gene (*Ddit4*) common to male and female datasets. (c) Schematic representation of the effect of GalCer on the function of *Ddit4* in brain.

Recently, there has been a surge in exploring gender differences in gene expression in relation to brain development and function. In this study, microarray analysis suggested significant sex differences, with little overlap between DEGs in male and female *Cln3*^*Δex7/8*^ mouse brains in response to treatment with Galcer. In Fig 9a, a Venn diagram depicts the number of unique and common DEGs in male and female *Cln3*^*Δex7/8*^ mouse brains. GalCer treatment of *Cln3*^*Δex7/8*^ mice was associated with distinct gene expression signatures for brain in each sex, with an overlap of a single gene, the DNA damage inducible transcript 4 (*Ddit4*) gene (Fig 9b). Additionally, a higher number of dysregulated genes in male versus female brain appeared. *Ddit4* is significantly downregulated following GalCer treatment in brain of female and male *Cln3*^*Δex7/8*^ mice (Fig 9b). DDIT4, an inhibitor of the mammalian target of rapamycin (mTOR) signaling, is a molecule involved in synaptic loss, neuronal atrophy, and depressive behavior [61]. The mTOR pathway has evolved as nutrient sensing in order to promote cell proliferation under adequate nutritional and environmental conditions. The activation of mTOR depends on the formation of two complexes, mTOR complex 1 (mTORC1) and mTOR complex 2 (mTORC2). mTORC1 is inactivated by rapamycin, but activated by growth factors, nutrients, and stress signals, as well as essential signaling pathways like PI3K and MAPK [62]. Hyperactivation of mTORC1 occurs in genetic models of Parkinson’s disease and Gaucher’s disease [63]. Activation of mTORC1 localized to the lysosomal membrane regulates cellular growth and homeostasis. Activation of mTORC1 impairs autophagy in Alzheimer disease and JNCL also known as CLN3 disease [64]. The beneficial activation of mTORC1 referred to here contradicts other studies claiming mTORC1 signaling attenuation, and not activation, to be beneficial in several neurodegenerative diseases. Results from multiple studies indicate that autophagy upregulation via mTORC1 inhibition attenuates neurodegenerative pathology in mouse models of Huntington’s disease, Alzheimer’s disease, and familial prion disease [65]. In the present study, GalCer may reduce apoptosis via downregulation of DDIT4. DDIT4 loses its ability to negatively regulate cell growth, proliferation and survival via inhibition of activity of mTORC1 (Fig 9c). As a result, activation of mTORC1 leads to increased cell growth and proliferation due to decreased autophagy and increased synthesis of lipids, ribosome biogenesis and translation [66]. Since mTOR plays a pivotal role in synaptic plasticity, inhibition of *Ddit4* by GalCer treatment may be a possible mechanism for the beneficial effect of GalCer in *Cln3*^*Δex7/8*^ mice.

## Conclusions

The data presented suggest that GalCer treatment affects a variety of signaling pathways accompanying orchestrated gene expression changes in *Cln3*^*Δex7/8*^ mouse brain. Results support the premise that GalCer affects complex regulation of cellular signaling pathways involved in this disease, and that this is gender-specific. Further studies may be required to understand how expression of these genes and their signaling pathways are correlated with the processes by which GalCer may be beneficial as a treatment for human CLN3 disease.

## Supporting information

S1 Fig Flowchart for the entire experimental procedure. This schematic figure illustrates the sequential steps of experiments and analyses applied in this study, indicating experimental groups and group sizes for each condition.
(PDF)

S1 Table Specific *mus musculus* primers used to confirm microarray results by qRT-PCR. (PDF)

S2 Table Upregulated genes in GalCer- *versus* vehicle-treated *Cln3*^*Δex7/8*^ male mouse brain. p-value < 0.05 with a cut-off ≥ + 1.3 fold-change.
(PDF)

S3 Table Downregulated genes in GalCer- *versus* vehicle-treated *Cln3*^*Δex7/8*^ male mouse brain. p-value < 0.05 with a cut-off ≥—1.3 fold-change.
(PDF)

S4 Table Differentially expressed genes (up- and down-regulated genes) in GalCer- *versus* vehicle-treated *Cln3*^*Δex7/8*^ female mouse brain. p-value < 0.05 with a cut-off ≥ ± 1.3 fold-change.
(PDF)

S5 Table Significant canonical pathways in (a) male and (b) female *Cln3*^*Δex7/8*^ mice. p-value < 0.05 is considered statistically significant.
(PDF)
