# Supplementary figures and images for: Sex differences in gene expression with galactosylceramide treatment in Cln3Δex7/8 mice
Source: PLoS One. 2020 Oct 2;15(10):e0239537. doi: 10.1371/journal.pone.0239537 (PMC7531864; doi:10.1371/journal.pone.0239537)

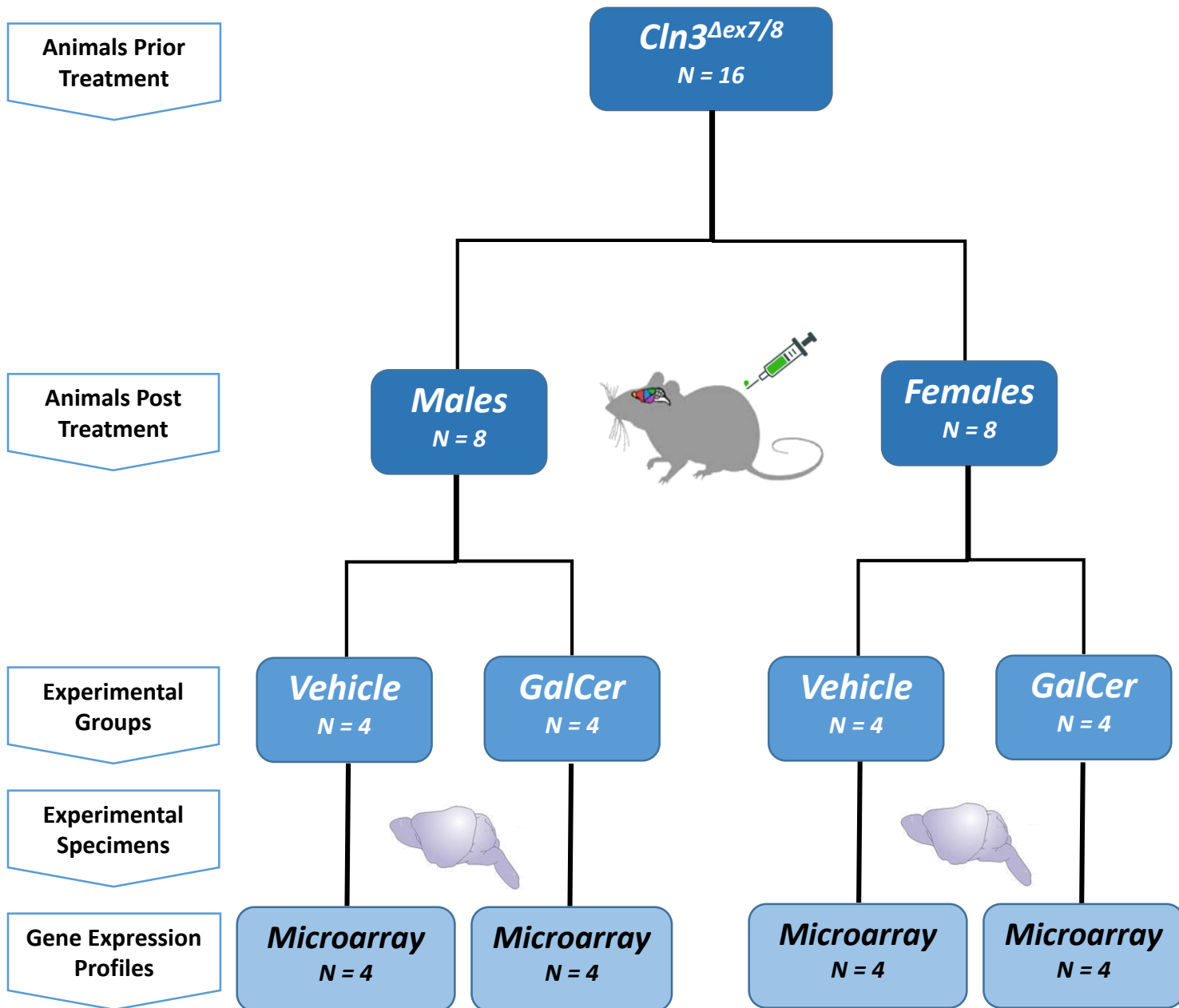

Supplement: S1 Fig — This schematic figure illustrates the sequential steps of experiments and analyses applied in this study, indicating experimental groups and group sizes for each condition. (PDF) [file pone.0239537.s001.pdf]
