# Supplementary material for: Sex differences in gene expression with galactosylceramide treatment in Cln3Δex7/8 mice
Source: PLoS One. 2020 Oct 2;15(10):e0239537. doi: 10.1371/journal.pone.0239537 (PMC7531864; doi:10.1371/journal.pone.0239537)
Supplement: S1 Table — (PDF) [file pone.0239537.s002.pdf]

Atp6v0a1-F<br>Atp6v0a1-R	TGAGGAAGAAGCATTTGGGAA<br>CCTCCTCTGTCGGTCCGTT
Braf-F<br>Braf-R	ACGTGTATGCGTTTGGGATT<br>TTTGAGGCACTCTGCCATTA
Dnm3-F<br>Dnm3-R	CAACGAAGGCTGACGATAAG<br>GGGAAAAGGAGGTAATGGG
Prkacb-F<br>Prkacb-R	GGGAGGAGAAAGATAGCC<br>AAACCAAACCAGGAGGAC
Gabbr1-F<br>Gabbr1-R	TCCACCAACAACAATGAGGA<br>GATGGCGCAGTTCAGAGAC
Grm1-F<br>Grm1-R	GGATCTGCACAGCCTGCAA<br>TCACTCCACTCGAGGTAACGG
C1qb-F<br>C1qb-R	TCTGGGAATCCACTGCTGTC<br>AGACCTCACCCCACTGTGTC
Fos-F<br>Fos-R	CGAAGGGAACGGAATAAGATG<br>GCTGCCAAAATAAACTCCAG
Prlr-F<br>Prlr-R	GGAAACATTCACCTGCTGGT<br>TATGGAAGTGTACTGCTTGCT
Fdft1-F<br>Fdft1-R	AGTGTGCCAACTCAATGGGTCTGT<br>TGTATCTGCCCCACACCTCCTGA
Gapdh-F<br>Gapdh-R	TGTTCCTACCCCCAATGTGT<br>AGTTGCTGTTGAAGTCGCAG

Additional file 2
