# Supplementary material for: Sex differences in gene expression with galactosylceramide treatment in Cln3Δex7/8 mice
Source: PLoS One. 2020 Oct 2;15(10):e0239537. doi: 10.1371/journal.pone.0239537 (PMC7531864; doi:10.1371/journal.pone.0239537)
Supplement: S2 Table — p-value < 0.05 with a cut-off ≥ + 1.3 fold-change. (PDF) [file pone.0239537.s003.pdf]

Gene Symbol	Gene Title	p-value	Fold-Change
**Atp6v0a1**	ATPase, H+ transporting, lysosomal V0 subunit A1	0.0033	1.545
**Kif5c**	kinesin family member 5C	0.0032	1.477
**Zic2**	zinc finger protein of the cerebellum 2	0.0099	1.474
**Slmap**	sarcolemma associated protein	0.0126	1.443
**Braf**	Braf transforming gene	0.0005	1.426
**Ptprs**	protein tyrosine phosphatase, receptor type, S	0.0083	1.425
**Mgat3**	mannoside acetylglucosaminyltransferase 3	0.0004	1.420
**Slc1a2**	solute carrier family 1 (glial high affinity glutamate transporter), member 2	0.0127	1.412
**Ncs1**	neuronal calcium sensor 1	0.0127	1.409
**Usp6nl**	USP6 N-terminal like	0.0033	1.397
**Cdk5r1**	cyclin-dependent kinase 5, regulatory subunit 1 (p35)	0.0172	1.387
**Dnm3**	dynamin 3	0.0005	1.386
**Prkacb**	protein kinase, cAMP dependent, catalytic, beta	0.0169	1.383
**Kif1b**	kinesin family member 1B	0.0004	1.377
**Gcc2**	GRIP and coiled-coil domain containing 2	0.0106	1.362
**Atrx**	alpha thalassemia/mental retardation syndrome X-linked homolog (human)	0.0003	1.358
**Kbtbd11**	kelch repeat and BTB (POZ) domain containing 11	0.0036	1.354
**Arnt2**	aryl hydrocarbon receptor nuclear translocator 2	0.0129	1.351
**Gabbr1**	gamma-aminobutyric acid (GABA) B receptor, 1	0.0147	1.344
**Eif4g1**	eukaryotic translation initiation factor 4, gamma 1	0.0020	1.343
**Hspa4**	heat shock protein 4	0.0151	1.342
**Taok2**	TAO kinase 2	0.0303	1.337
**Dpp6**	dipeptidylpeptidase 6	0.0011	1.337
**Jak1**	Janus kinase 1	0.0103	1.336
**Acly**	ATP citrate lyase	0.0007	1.335
**Map1b**	microtubule-associated protein 1B	0.0084	1.332
**Sdf4**	stromal cell derived factor 4	0.0019	1.332
**Fgfr2**	fibroblast growth factor receptor 2	0.0004	1.332
**Sema7a**	sema domain, immunoglobulin domain (Ig), and GPI membrane anchor, (semaphorin) 7A	0.0010	1.331
**Dgkd**	diacylglycerol kinase, delta	0.0004	1.328
**Nav3**	neuron navigator 3	0.0107	1.326
**Atp2b2**	ATPase, Ca++ transporting, plasma membrane 2	0.0025	1.326
**Extl3**	exostoses (multiple)-like 3	0.0028	1.324
**Fam168a**	**family with sequence similarity 168, member A**	**0.0005**	**1.324**
**Kcnq2**	potassium voltage-gated channel, subfamily Q, member 2	0.0293	1.322
**Rfx3**	regulatory factor X, 3 (influences HLA class II expression)	0.0230	1.322
**Cdk12**	cyclin-dependent kinase 12	0.0187	1.315
**Ndst1**	N-deacetylase/N-sulfotransferase (heparan glucosaminyl) 1	0.0171	1.315
**Copg2os2**	coatomer protein complex, subunit gamma 2, opposite strand 2	0.0480	1.314
**Neb**	nebulin	0.0094	1.310
**Setd5**	SET domain containing 5	0.0026	1.310
**Grm1**	glutamate receptor, metabotropic 1	0.0036	1.309
**Dlg4**	discs, large homolog 4 (Drosophila)	0.0220	1.308
**Nr3c1**	nuclear receptor subfamily 3, group C, member 1	0.0337	1.308
**Dnmt3a**	DNA methyltransferase 3A	0.0058	1.307
**Top2b**	topoisomerase (DNA) II beta	0.0293	1.306
**Car8**	carbonic anhydrase 8	0.0022	1.305
**Slc35f1**	solute carrier family 35, member F1	0.0174	1.302
**Kif5a**	kinesin family member 5A	0.0084	1.301

Additional file 3
