# Supplementary material for: Sex differences in gene expression with galactosylceramide treatment in Cln3Δex7/8 mice
Source: PLoS One. 2020 Oct 2;15(10):e0239537. doi: 10.1371/journal.pone.0239537 (PMC7531864; doi:10.1371/journal.pone.0239537)
Supplement: S3 Table — p-value < 0.05 with a cut-off ≥—1.3 fold-change. (PDF) [file pone.0239537.s004.pdf]

Gene Symbol	Gene Title	p-value	Fold-Change
**Sox2ot**	SOX2 overlapping transcript (non-protein coding)	0.0061	-1.302
**Cox15**	cytochrome c oxidase assembly protein 15	0.0236	-1.303
**Ddit4**	DNA-damage-inducible transcript 4	0.0280	-1.308
**C1qb**	complement component 1, q subcomponent, beta polypeptide	0.0302	-1.310
**Mobp**	myelin-associated oligodendrocytic basic protein	0.0318	-1.312
**Meg3**	maternally expressed 3	0.0486	-1.326
**Snhg11**	small nucleolar RNA host gene 11	0.0066	-1.331
**Rsad2**	radical S-adenosyl methionine domain containing 2	0.0042	-1.354
**Mapk8**	mitogen-activated protein kinase 8	0.0077	-1.355
**Vcp**	valosin containing protein	0.0126	-1.356
**Fubp1**	far upstream element (FUSE) binding protein 1	0.0202	-1.370
**Ogt**	O-linked N-acetylglucosamine (GlcNAc) transferase (UDP-N-acetylglucosamine:polypeptide)	0.0072	-1.373
**Gtf3c2**	general transcription factor IIIC, polypeptide 2, beta	0.0084	-1.381
**Araf**	v-raf murine sarcoma 3611 viral oncogene homolog	0.0078	-1.425
**Miat**	myocardial infarction associated transcript (non-protein coding)	0.0089	-1.511
**Ltbp3**	latent transforming growth factor beta binding protein 3	0.0124	-1.631
**Igk-V28**	immunoglobulin kappa chain variable 28 (V28)	0.0304	-1.773

Additional file 4
