# Supplementary material for: Sex differences in gene expression with galactosylceramide treatment in Cln3Δex7/8 mice
Source: PLoS One. 2020 Oct 2;15(10):e0239537. doi: 10.1371/journal.pone.0239537 (PMC7531864; doi:10.1371/journal.pone.0239537)
Supplement: S4 Table — p-value < 0.05 with a cut-off ≥ ± 1.3 fold-change. (PDF) [file pone.0239537.s005.pdf]

a

Gene Symbol	Gene Title	p-value	Fold-Change
**Rbmx**	RNA binding motif protein, X chromosome	0.036	1.49
**Cox7c**	cytochrome c oxidase subunit VIIc	0.032	1.47
**Cx3cl1**	chemokine (C-X3-C motif) ligand 1	0.041	1.47
**Rps24**	ribosomal protein S24	0.044	1.43
**Meis2**	Meis homeobox 2	0.042	1.40
**Gas5**	growth arrest specific 5	0.019	1.38
**Usp40**	ubiquitin specific peptidase 40	0.001	1.38
**Iigp1**	interferon inducible GTPase 1	0.028	1.35
**Ccdc58**	coiled-coil domain containing 58	0.019	1.34
**Mex3b**	mex3 homolog B (C. elegans)	0.011	1.34
**Xist**	inactive X specific transcripts	0.004	1.32
**Scly**	selenocysteine lyase	0.047	1.32
**Zc3h4**	zinc finger CCCH-type containing 4	0.016	1.32
**Herc2**	hect (homologous to the E6-AP (UBE3A) carboxyl terminus) domain and RCC1 (CHC1)-like do	0.001	1.31
**Zyg11b**	zyg-ll family member B, cell cycle regulator	0.027	1.31
**Snord89**	small nucleolar RNA, C/D box 89	0.014	1.30

b

Gene Symbol	Gene Title	p-value	Fold-Change
**Pnliprp1**	pancreatic lipase related protein 1	0.008	-1.31
**Pax3**	paired box 3	0.010	-1.31
**Fdft1**	farnesyl diphosphate farnesyl transferase 1	0.010	-1.31
**Slc22a29**	solute carrier family 22. member 29	0.022	-1.31
**Six4**	sine oculis-related homeobox 4	0.049	-1.32
**Ddit4**	DNA-damage-inducible transcript 4	0.012	-1.34
**Klf2**	Kruppel-like factor 2 (lung)	0.032	-1.37
**Zfp36**	zinc finger protein 36	0.016	-1.39
**Prlr**	prolactin receptor	0.011	-1.39
**Apold1**	apolipoprotein L domain containing 1	0.010	-1.42
**Dusp1**	dual specificity phosphatase 1	0.013	-1.67
**Fos**	FBJ osteosarcoma oncogene	0.027	-2.05
**Cyr61**	cysteine rich protein 61	0.014	-2.32

Additional file 5
