# Supplementary material for: Sex differences in gene expression with galactosylceramide treatment in Cln3Δex7/8 mice
Source: PLoS One. 2020 Oct 2;15(10):e0239537. doi: 10.1371/journal.pone.0239537 (PMC7531864; doi:10.1371/journal.pone.0239537)
Supplement: S5 Table — p-value < 0.05 is considered statistically significant. (PDF) [file pone.0239537.s006.pdf]

a

Pathway Name	Enrichment Score	p-value
**Long-term potentiation**	7.657	0.0005
**Pancreatic cancer**	7.163	0.0008
**EGFR tyrosine kinase inhibitor resistance**	6.917	0.0010
**Progesterone-mediated oocyte maturation**	6.556	0.0014
**Endocrine resistance**	6.349	0.0017
**Cocaine addiction**	6.062	0.0023
**Taste transduction**	5.772	0.0031
**Long-term depression**	5.319	0.0049
**Renal cell carcinoma**	5.016	0.0066
**Glycosaminoglycan biosynthesis**	4.903	0.0074
**Colorectal cancer**	4.896	0.0075
**ErbB signaling pathway**	4.501	0.0111
**Prion diseases**	4.262	0.0141
**Estrogen signaling pathway**	4.162	0.0156
**Prostate cancer**	4.022	0.0179
**Bladder cancer**	3.885	0.0205
**Endocrine and other factor-regulated calcium reabsorption**	3.315	0.0363
**Endometrial cancer**	3.214	0.0402
**Synaptic vesicle cycle**	3.059	0.0469

b

Pathway Name	Enrichment Score	p-value
**Prolactin signaling pathway**	4.211	0.015
**Selenocompound metabolism**	3.131	0.044
**Steroid biosynthesis**	3.022	0.049

Additional file 6
